# Supplementary material for: LTA4H extensively associates with mRNAs and lncRNAs indicative of its novel regulatory targets
Source: PeerJ. 2023 Mar 10;11:e14875. doi: 10.7717/peerj.14875 (PMC10010175; doi:10.7717/peerj.14875)
Supplement: File S2 [file peerj-11-14875-s005.zip › Raw data files/Quality_control/FastqcView/LTA4H_input_2_fastqc/fastqc_report.html]

E743-input-2-mRNA\_all.fq.clean\_fq\_reverse FastQC Report


FastQC Report

Mon 16 Oct 2017  
E743-input-2-mRNA\_all.fq.clean\_fq\_reverse

## Summary

- Basic Statistics
- Per base sequence quality
- Per sequence quality scores
- Per base sequence content
- Per base GC content
- Per sequence GC content
- Per base N content
- Sequence Length Distribution
- Sequence Duplication Levels
- Overrepresented sequences
- Kmer Content

## Basic Statistics

| Measure | Value |
| --- | --- |
| Filename | E743-input-2-mRNA\_all.fq.clean\_fq\_reverse |
| File type | Conventional base calls |
| Encoding | Sanger / Illumina 1.9 |
| Total Sequences | 38611163 |
| Sequence length | 16-147 |
| %GC | 45 |

## Per base sequence quality

## Per sequence quality scores

## Per base sequence content

## Per base GC content

## Per sequence GC content

## Per base N content

## Sequence Length Distribution

## Sequence Duplication Levels

## Overrepresented sequences

| Sequence | Count | Percentage | Possible Source |
| --- | --- | --- | --- |
| TGTGTGCCTAATACATGCATGTTGAACGGGATGTAGCAATACATTCAGTA | 89743 | 0.23242760131312284 | No Hit |
| CTACGGGGTCTGACGCTCAGTGGAACGAAAACTCACGTTAAGGGATTTTG | 74379 | 0.19263600011219553 | No Hit |
| GTGGGCGTTGGAATATTGATGAGAGCTGCTCTTAGTACGAGAGGACCGGA | 54910 | 0.14221275852271015 | No Hit |
| GGCCGGTGTTCTTCAGGATCTGCTTGCTCACCATGGTTGTGGCCATTATC | 48680 | 0.12607752840804096 | No Hit |
| GATTCGAAGTGAGAATCTTCGACGCCTATTGGGAAAGGTTTCCTGGGCAA | 45389 | 0.11755408662515554 | No Hit |
| ACGGTGAAGAGACATGAGAGGTGTAGAATAAGTGGGAGGCCCCCGGCGCC | 40079 | 0.10380158712132033 | No Hit |

## Kmer Content

No overrepresented Kmers

Produced by FastQC (version 0.9.5)
